# Bis(tetra­phenyl­phospho­nium) bis­[*N*-(octyl­sulfon­yl)dithio­carbimato(2–)-κ^2^ *S*,*S*′]­nickelate(II)

**DOI:** 10.1107/S1600536807065014

**Published:** 2007-12-12

**Authors:** Leandro M. G. Cunha, Mayura M. M. Rubinger, Marcelo R. L. Oliveira, Jose R. Sabino

**Affiliations:** a Departamento de Química, UFV, 36570-000 Viçosa, MG, Brazil; b Instituto de Física, UFG, Caixa Postal 131, 74001-970 Goiânia, Brazil

## Abstract

The Ni atom in the title complex, (C_24_H_20_P)_2_[Ni(C_9_H_17_NO_2_S_3_)_2_], lies on a twofold axis within a square-planar geometry defined by four S atoms derived from two dithio­carbimate dianions, each forming a four-membered chelate ring. A small distortion, described by a deviation of the Ni^II^ atom by 0.083 (1) Å from the plane through the four S atoms, and also by the torsion angles about the Ni—S bonds, implies a folded conformation for the chelate ring.

## Related literature

The title complex is a new member of the class of Ni complexes with general formula [Ni(*R*—SO_2_N=CS_2_)_2_]^2−^ (Hummel *et al.*, 1989 ▶; Franca *et al.*, 2006 ▶; Oliveira *et al.*, 1997 ▶, 1999 ▶, 2003 ▶). The literature describes only two other complexes of this class having tetra­phenyl­phospho­nium as counter-ion (Hummel & Korn, 1989 ▶; Allen, 2002 ▶). For other related literature, see: Hogarth (2005 ▶); Vogel (1966 ▶); Cremer & Pople (1975 ▶).
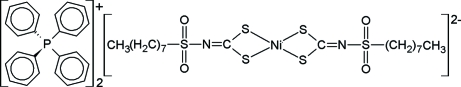


## Experimental

### 

#### Crystal data

(C_24_H_20_P)_2_[Ni(C_9_H_17_NO_2_S_3_)_2_]
*M* *_r_* = 1272.32
Monoclinic, 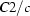

*a* = 29.113 (4) Å
*b* = 10.425 (2) Å
*c* = 22.966 (3) Å
β = 115.50 (1)°
*V* = 6291.3 (18) Å^3^
*Z* = 4
Cu *K*α radiation
μ = 3.17 mm^−1^
*T* = 297 (2) K
0.16 × 0.16 × 0.08 mm

#### Data collection

Enraf–Nonius CAD-4 diffractometer
Absorption correction: Gaussian (Spek, 2003 ▶) *T*_min_ = 0.629, *T*_max_ = 0.787
11798 measured reflections
5696 independent reflections
3927 reflections with *I* > 2σ(*I*)
*R*_int_ = 0.079
2 standard reflections frequency: 120 min intensity decay: 1%

#### Refinement

*R*[*F* ^2^ > 2σ(*F* ^2^)] = 0.061
*wR*(*F* ^2^) = 0.208
*S* = 1.05
5696 reflections
367 parameters
5 restraints
H-atom parameters constrained
Δρ_max_ = 0.59 e Å^−3^
Δρ_min_ = −0.61 e Å^−3^

### 

Data collection: *CAD-4-PC* (Enraf–Nonius, 1993 ▶); cell refinement: *CAD-4-PC*; data reduction: *XCAD4* (Harms & Wocadlo, 1995 ▶); program(s) used to solve structure: *SHELXS97* (Sheldrick, 1997 ▶); program(s) used to refine structure: *SHELXL97* (Sheldrick, 1997 ▶); molecular graphics: *PLATON* (Spek, 2003 ▶); software used to prepare material for publication: *WinGX* (Farrugia, 1999 ▶).

## Supplementary Material

Crystal structure: contains datablocks global, I. DOI: 10.1107/S1600536807065014/tk2230sup1.cif

Structure factors: contains datablocks I. DOI: 10.1107/S1600536807065014/tk2230Isup2.hkl

Additional supplementary materials:  crystallographic information; 3D view; checkCIF report

## Figures and Tables

**Table:** 

Ni—S1	2.2048 (12)
Ni—S2	2.2075 (11)

**Table:** 

S1—Ni—S2	78.52 (4)

**Table:** 

S2^i^—Ni—S1—C1	169.45 (15)
S1^i^—Ni—S2—C1	−169.41 (15)
C2—S3—N1—C1	−63.9 (4)

Symmetry code: (i) 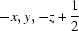.

**Table 2:** Hydrogen-bond geometry (Å, °)

*D*—H⋯*A*	*D*—H	H⋯*A*	*D*⋯*A*	*D*—H⋯*A*
C2—H2*B*⋯S2	0.97	2.83	3.490 (5)	126
C13—H13⋯O2^ii^	0.93	2.58	3.276 (6)	132

Symmetry code: (ii) 
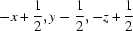
.

**Fig. 1.**
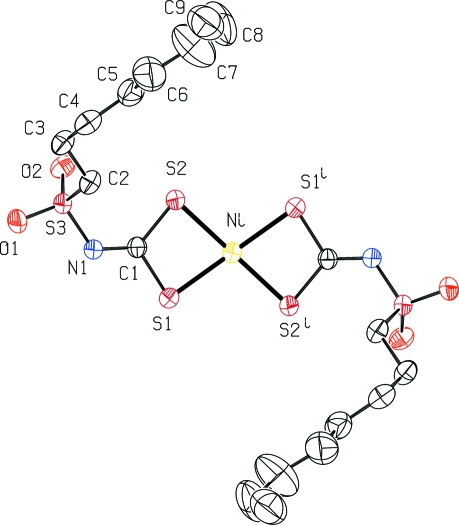
View of the dianion in (I) with 30% probability displacement ellipsoids showing atom labelling scheme. Symemtry operation (i): -x, y, -z + 1/2.

**Fig. 2.**
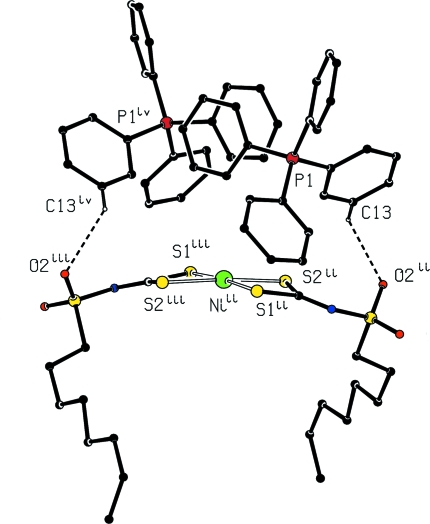
H-bonding in (I). The b axis is oriented upward and the a axis points to the right. Symmetry operation (iii): x+1/2, y - 1/2, z; (iv) -x+1, y, -z + 1/2. Only the hydrogen atoms participating in the interactions are shown.

## supplementary crystallographic information

### Comment

We became interested in the syntheses and characterization of nickel(II) dithiocarbimates complexes due to their similarity with the dithiocarbamates, which have been used as molecular precursors for various nickel sulfides by MOCVD techniques (Hogarth, 2005). Some anionic nickel-dithiocarbimato complexes with general formula [Ni(RSO_2_N=CS_2_)_2_]^2-^ (*R* = aryl or alkyl groups) have had their structures determined by X-ray diffraction techniques (Oliveira *et al.*, 1997; Oliveira *et al.*, 1999; Oliveira *et al.*, 2003). However, only two of these complexes have the tetraphenylphosphonium as the counterion (Hummel & Korn, 1989) and only two were aliphatic (Oliveira *et al.*, 1997; Franca *et al.*, 2006). Variations in the counter-ions and in the *R* group can be important to modulate the volatility of these compounds favouring their application in MOCVD techniques. The title complex, (I), which is quite stable under ambient conditions, comprises a complex dianion and two tetraphenylphosphonium cations, with the formula (Ph_4_P)_2_(Ni(C_8_H_17_SO_2_N=CS_2_)_2_)^2-^, Figs 1 & 2.

The Ni^II^ ion is located in a twofold axis of symmetry being coordinated by four sulfur atoms from the dithiocarbimate dianion in a square planar coordination environment, Fig. 1 & Table 1. The Ni centre is located at 0.083 (1) Å out of the plane through the 4 S atoms. The resultant 4-membered Ni/S1/C1/S2 chelate ring shows a folded conformation [C&P Q(2) of 0.113 (3) Å; (Cremer & Pople, 1975)], giving the torsion angles S1^i^—Ni—S2—C1 and S2^i^—Ni—S1—C1 of 169.5 (2)° and -169.4 (2)°, respectively [symmetry code: (i) -*x*, *y*, -*z* + 1/2]. These values are outside the range from 174° to 180° observed in the related structures, with the smaller value found in (C_14_H_10_N_2_NiO_4_S_6_)^2-^.2(C_24_H_20_P)^+^ (Hummel & Korn, 1989), showing an higher distortion of the chelate ring in (I). This might be caused by the requirements of the packing of the counterion.

The conformation of (I) is stabilized by a weak intra-molecular H-bond of type C2–H2B···S2 (Table 2), which defines the torsion angle C1–N1–S3–C2 of -63.9 (4)°. Due to the flexibility of the long C chain, disorder was evident [see Experimental] so that the only bond distances determined reliably were C2—C3 [1.517 (7) Å] and C3—C4 [1.507 (7) Å]. The other C—C bonds were restrained to 1.54 Å and the chain conformation might be described, starting from the torsion angle about the C2–C3 bond, as: *trans*, *gauche*, *trans*, *trans*, *cis*, respectively. The actual torsion angles deviate from the ideal 0°, 60° and 180° due to repulsion due to the neighbouring molecules' C chains.

### Experimental

The octanesulfonamide was prepared from octanesulfonyl chloride in a similar procedure as described elsewhere (Vogel, 1966). Potassium *N*-(octylsulfonyl)dithiocarbimate was prepared from the sulfonamide using procedures described in the literature for analogous compounds Complex (I) was prepared in 1:1 (10 ml) methanol:water mixture from NiCl_2_.6H_2_O (1.0 mmol), potassium *N*-(octylsulfonyl)dithiocarbimate dihydrate (1.0 mmol) and tetraphenylphosphonium bromide (2 mmol). The reaction mixture was stirred for 1 h at room temperature. The green solid obtained was filtered, washed with distilled water and dried under reduced pressure for 1 day. Suitable crystals of (I) were obtained by slow evaporation of the solvent water/methanol (1:1 *v*/*v*); m. pt. 427.5–429.1 K. Analysis found: C 62.43, H 5.81, N 2.42, Ni 4.59; C_66_H_74_N_2_NiO_4_P_2_S_6_ requires: C 62.30, H 5.86, N 2.20, Ni 4.61%. IR (most important bands, cm^-1^): 1398 ν(C=N); 1268 ν*_asym_*(SO_2_); 1123 ν*_sym_*(SO_2_); 936 ν*_asym_*(CS_2_) and 381 ν(NiS).

### Refinement

All H atoms were positioned geometrically and allowed to ride on their parent atoms with C—H distances in the range 0.93–0.97 Å, and with *U*_iso_(H) = 1.5 *U*_eq_(C) for methyl-H atoms and *U*_iso_(H) = 1.2 *U*_eq_(C) for other atoms. The bond distances C4–C5, C5–C6, C6–C7, C7–C8 and C8–C9 were restrained to 1.54 Å. The atoms C5 to C9 are very disordered and any attempt to model this disorder over multiple sites was not reliable.

### Crystal data

**Table:**

(C_24_H_20_P)_2_[Ni(C_9_H_17_NO_2_S_3_)_2_]	*F*_000_ = 2680
*M**_r_* = 1272.32	*D*_x_ = 1.343 Mg m^−^^3^
Monoclinic, *C*2/*c*	Melting point: 428 K
Hall symbol: -C 2yc	Cu *K*α radiation λ = 1.54180 Å
*a* = 29.113 (4) Å	Cell parameters from 25 reflections
*b* = 10.425 (2) Å	θ = 16.2–30.1º
*c* = 22.966 (3) Å	µ = 3.17 mm^−^^1^
β = 115.50 (1)º	*T* = 297 (2) K
*V* = 6291.3 (18) Å^3^	Prism, dark-yellow
*Z* = 4	0.16 × 0.16 × 0.08 mm

### Data collection

**Table:**

Enraf–Nonius CAD-4 diffractometer	*R*_int_ = 0.079
Radiation source: fine-focus sealed tube	θ_max_ = 68º
Monochromator: graphite	θ_min_ = 3.4º
*T* = 298(2) K	*h* = −34→34
non–profiled ω/2θ scans	*k* = −12→12
Absorption correction: Gaussian(Spek, 2003)	*l* = −18→27
*T*_min_ = 0.629, *T*_max_ = 0.787	2 standard reflections
11798 measured reflections	every 120 min
5696 independent reflections	intensity decay: 1%
3927 reflections with *I* > 2σ(*I*)	

### Refinement

**Table:**

Refinement on *F*^2^	Hydrogen site location: inferred from neighbouring sites
Least-squares matrix: full	H-atom parameters constrained
*R*[*F*^2^ > 2σ(*F*^2^)] = 0.061	*w* = 1/[σ^2^(*F*_o_^2^) + (0.1159*P*)^2^ + 8.6294*P*] where *P* = (*F*_o_^2^ + 2*F*_c_^2^)/3
*wR*(*F*^2^) = 0.208	(Δ/σ)_max_ < 0.001
*S* = 1.05	Δρ_max_ = 0.59 e Å^−^^3^
5696 reflections	Δρ_min_ = −0.61 e Å^−^^3^
367 parameters	Extinction correction: SHELXL97, Fc^*^=kFc[1+0.001xFc^2^λ^3^/sin(2θ)]^-1/4^
5 restraints	Extinction coefficient: 0.00064 (8)
Secondary atom site location: difference Fourier map	

### Special details

**Table:**

Geometry. All e.s.d.'s (except the e.s.d. in the dihedral angle between two l.s. planes) are estimated using the full covariance matrix. The cell e.s.d.'s are taken into account individually in the estimation of e.s.d.'s in distances, angles and torsion angles; correlations between e.s.d.'s in cell parameters are only used when they are defined by crystal symmetry. An approximate (isotropic) treatment of cell e.s.d.'s is used for estimating e.s.d.'s involving l.s. planes.

### Fractional atomic coordinates and isotropic or equivalent isotropic displacement parameters (Å^2^)

**Table:**

	*x*	*y*	*z*	*U*_iso_*/*U*_eq_	
Ni	0	0.82980 (10)	0.25	0.0545 (3)	
S1	0.07020 (4)	0.82179 (13)	0.34078 (6)	0.0654 (4)	
S2	0.05891 (4)	0.82194 (12)	0.21372 (5)	0.0622 (3)	
S3	0.18146 (4)	0.73175 (12)	0.27711 (5)	0.0574 (3)	
O2	0.17880 (14)	0.8302 (3)	0.23259 (18)	0.0748 (9)	
O1	0.23151 (12)	0.6954 (4)	0.32300 (17)	0.0849 (11)	
N1	0.15016 (13)	0.7668 (4)	0.31917 (17)	0.0590 (9)	
C1	0.10265 (16)	0.7992 (4)	0.2937 (2)	0.0555 (10)	
C2	0.15171 (18)	0.5925 (5)	0.2332 (3)	0.0697 (12)	
H2A	0.1498	0.5287	0.2628	0.084*	
H2B	0.1172	0.6136	0.2028	0.084*	
C3	0.1798 (2)	0.5359 (5)	0.1968 (3)	0.0793 (15)	
H3A	0.1839	0.6016	0.1695	0.095*	
H3B	0.2134	0.509	0.2275	0.095*	
C4	0.1522 (2)	0.4229 (6)	0.1558 (3)	0.0872 (16)	
H4A	0.1425	0.3646	0.1816	0.105*	
H4B	0.1752	0.3773	0.1426	0.105*	
C5	0.1043 (2)	0.4607 (7)	0.0953 (3)	0.113 (2)	
H5A	0.1126	0.5196	0.0686	0.136*	
H5B	0.0792	0.5003	0.1069	0.136*	
C6	0.0845 (4)	0.3320 (8)	0.0602 (5)	0.186 (5)	
H6A	0.109	0.2948	0.0468	0.224*	
H6B	0.0783	0.2716	0.0881	0.224*	
C7	0.0349 (5)	0.3651 (11)	0.0013 (6)	0.271 (9)	
H7A	0.0424	0.4276	−0.0246	0.325*	
H7B	0.012	0.4056	0.0164	0.325*	
C8	0.0069 (5)	0.2514 (11)	−0.0422 (6)	0.241 (8)	
H8A	−0.0262	0.2494	−0.0414	0.289*	
H8B	0.0005	0.2784	−0.0855	0.289*	
C9	0.0228 (4)	0.1100 (10)	−0.0385 (5)	0.191 (5)	
H9A	−0.0051	0.0606	−0.0687	0.286*	
H9B	0.0321	0.0783	0.0044	0.286*	
H9C	0.0513	0.1029	−0.0488	0.286*	
P1	0.36685 (3)	0.78525 (10)	0.07711 (5)	0.0464 (3)	
C21	0.35663 (14)	0.6605 (4)	0.0188 (2)	0.0511 (9)	
C22	0.32109 (16)	0.5653 (4)	0.0083 (2)	0.0635 (11)	
H22	0.3055	0.5556	0.0359	0.076*	
C23	0.3090 (2)	0.4840 (5)	−0.0440 (3)	0.0807 (15)	
H23	0.285	0.4197	−0.0514	0.097*	
C24	0.3315 (2)	0.4963 (6)	−0.0847 (3)	0.0868 (17)	
H24	0.3226	0.4411	−0.1197	0.104*	
C25	0.3678 (2)	0.5917 (6)	−0.0740 (2)	0.0788 (15)	
H25	0.3834	0.6006	−0.1016	0.095*	
C26	0.38034 (18)	0.6729 (5)	−0.0218 (2)	0.0664 (12)	
H26	0.4048	0.7361	−0.0138	0.08*	
C31	0.43463 (14)	0.8089 (4)	0.12143 (19)	0.0496 (9)	
C32	0.46464 (16)	0.7003 (4)	0.1410 (2)	0.0606 (11)	
H32	0.4504	0.6194	0.1282	0.073*	
C33	0.51645 (17)	0.7136 (5)	0.1802 (2)	0.0703 (13)	
H33	0.5369	0.641	0.1939	0.084*	
C34	0.53720 (17)	0.8314 (5)	0.1985 (3)	0.0727 (14)	
H34	0.5718	0.8387	0.2253	0.087*	
C35	0.50824 (18)	0.9399 (5)	0.1782 (3)	0.0746 (14)	
H35	0.5231	1.0203	0.1906	0.09*	
C36	0.45635 (16)	0.9291 (4)	0.1388 (2)	0.0621 (11)	
H36	0.4364	1.0023	0.1242	0.075*	
C41	0.33391 (14)	0.9246 (4)	0.03316 (18)	0.0474 (9)	
C42	0.30224 (14)	0.9135 (4)	−0.03291 (19)	0.0520 (9)	
H42	0.299	0.835	−0.0535	0.062*	
C43	0.27604 (15)	1.0182 (4)	−0.0673 (2)	0.0556 (10)	
H43	0.2546	1.0099	−0.111	0.067*	
C44	0.28115 (16)	1.1355 (5)	−0.0377 (2)	0.0618 (11)	
H44	0.2638	1.2066	−0.0615	0.074*	
C45	0.31226 (17)	1.1474 (4)	0.0278 (2)	0.0634 (11)	
H45	0.3157	1.2266	0.0479	0.076*	
C46	0.33794 (15)	1.0433 (4)	0.0630 (2)	0.0546 (10)	
H46	0.3582	1.0517	0.1071	0.065*	
C11	0.34119 (14)	0.7416 (4)	0.13264 (19)	0.0488 (9)	
C12	0.36240 (16)	0.6398 (4)	0.1756 (2)	0.0601 (11)	
H12	0.3891	0.5926	0.1744	0.072*	
C13	0.34362 (17)	0.6096 (5)	0.2196 (2)	0.0639 (11)	
H13	0.3582	0.5432	0.2488	0.077*	
C14	0.30335 (18)	0.6772 (5)	0.2207 (2)	0.0675 (13)	
H14	0.2907	0.6549	0.2503	0.081*	
C15	0.28174 (17)	0.7762 (5)	0.1792 (2)	0.0649 (12)	
H15	0.2547	0.8216	0.1806	0.078*	
C16	0.30044 (16)	0.8092 (4)	0.1344 (2)	0.0589 (11)	
H16	0.2857	0.8763	0.1057	0.071*	

### Atomic displacement parameters (Å^2^)

**Table:**

	*U*^11^	*U*^22^	*U*^33^	*U*^12^	*U*^13^	*U*^23^
Ni	0.0469 (5)	0.0568 (6)	0.0611 (6)	0	0.0243 (5)	0
S1	0.0547 (6)	0.0852 (9)	0.0590 (7)	0.0010 (5)	0.0271 (5)	−0.0024 (6)
S2	0.0493 (6)	0.0812 (8)	0.0548 (6)	0.0051 (5)	0.0213 (5)	0.0067 (5)
S3	0.0461 (5)	0.0688 (7)	0.0559 (6)	0.0026 (4)	0.0206 (5)	−0.0007 (5)
O2	0.088 (2)	0.065 (2)	0.087 (2)	−0.0014 (17)	0.054 (2)	0.0053 (17)
O1	0.0511 (18)	0.126 (3)	0.069 (2)	0.0167 (18)	0.0180 (16)	−0.006 (2)
N1	0.0489 (18)	0.076 (2)	0.0522 (19)	0.0010 (17)	0.0215 (15)	0.0001 (18)
C1	0.052 (2)	0.054 (2)	0.059 (2)	−0.0034 (18)	0.0225 (19)	−0.0018 (19)
C2	0.065 (3)	0.066 (3)	0.086 (3)	0.000 (2)	0.041 (3)	−0.003 (3)
C3	0.075 (3)	0.080 (4)	0.097 (4)	−0.006 (3)	0.051 (3)	−0.011 (3)
C4	0.103 (4)	0.078 (4)	0.104 (4)	−0.005 (3)	0.066 (4)	−0.006 (3)
C5	0.102 (5)	0.131 (6)	0.117 (6)	−0.024 (4)	0.058 (5)	−0.019 (5)
C6	0.151 (9)	0.249 (14)	0.147 (9)	0.014 (9)	0.054 (7)	−0.079 (9)
C7	0.259 (18)	0.29 (2)	0.175 (12)	−0.043 (14)	0.007 (13)	−0.060 (14)
C8	0.154 (10)	0.306 (19)	0.185 (12)	0.055 (12)	0.000 (9)	−0.129 (13)
C9	0.182 (11)	0.218 (14)	0.141 (9)	−0.021 (10)	0.040 (8)	0.020 (9)
P1	0.0402 (5)	0.0469 (6)	0.0489 (6)	0.0006 (4)	0.0164 (4)	0.0005 (4)
C21	0.0459 (19)	0.049 (2)	0.055 (2)	0.0075 (16)	0.0184 (17)	0.0009 (18)
C22	0.056 (2)	0.051 (2)	0.079 (3)	−0.0062 (19)	0.025 (2)	−0.004 (2)
C23	0.070 (3)	0.060 (3)	0.094 (4)	−0.005 (2)	0.018 (3)	−0.019 (3)
C24	0.087 (4)	0.076 (4)	0.083 (4)	0.015 (3)	0.022 (3)	−0.028 (3)
C25	0.082 (3)	0.090 (4)	0.060 (3)	0.011 (3)	0.027 (3)	−0.013 (3)
C26	0.062 (3)	0.070 (3)	0.067 (3)	−0.001 (2)	0.028 (2)	−0.005 (2)
C31	0.0409 (19)	0.055 (2)	0.050 (2)	−0.0031 (16)	0.0177 (17)	0.0007 (18)
C32	0.048 (2)	0.061 (3)	0.069 (3)	0.0011 (19)	0.021 (2)	0.002 (2)
C33	0.047 (2)	0.083 (3)	0.078 (3)	0.008 (2)	0.023 (2)	0.014 (3)
C34	0.043 (2)	0.096 (4)	0.073 (3)	−0.010 (2)	0.019 (2)	0.007 (3)
C35	0.056 (2)	0.076 (3)	0.089 (4)	−0.020 (2)	0.028 (2)	−0.002 (3)
C36	0.049 (2)	0.062 (3)	0.075 (3)	−0.0077 (19)	0.027 (2)	0.003 (2)
C41	0.0435 (18)	0.049 (2)	0.047 (2)	0.0023 (16)	0.0167 (16)	0.0017 (17)
C42	0.047 (2)	0.052 (2)	0.051 (2)	−0.0022 (17)	0.0163 (17)	−0.0052 (18)
C43	0.052 (2)	0.060 (3)	0.050 (2)	0.0046 (19)	0.0181 (18)	0.004 (2)
C44	0.058 (2)	0.057 (3)	0.066 (3)	0.013 (2)	0.023 (2)	0.009 (2)
C45	0.068 (3)	0.050 (2)	0.067 (3)	0.008 (2)	0.024 (2)	−0.007 (2)
C46	0.053 (2)	0.054 (2)	0.053 (2)	0.0023 (18)	0.0191 (18)	−0.0032 (19)
C11	0.0417 (19)	0.049 (2)	0.051 (2)	−0.0034 (16)	0.0154 (17)	−0.0023 (17)
C12	0.054 (2)	0.057 (2)	0.067 (3)	0.0031 (19)	0.023 (2)	0.007 (2)
C13	0.061 (2)	0.065 (3)	0.063 (3)	−0.008 (2)	0.024 (2)	0.007 (2)
C14	0.063 (3)	0.084 (3)	0.060 (3)	−0.021 (2)	0.030 (2)	−0.008 (2)
C15	0.056 (2)	0.075 (3)	0.073 (3)	0.002 (2)	0.037 (2)	−0.002 (3)
C16	0.048 (2)	0.064 (3)	0.063 (3)	0.0029 (18)	0.0214 (19)	0.004 (2)

### Geometric parameters (Å, °)

**Table:**

Ni—S1	2.2048 (12)	C22—H22	0.93
Ni—S1^i^	2.2048 (12)	C23—C24	1.359 (8)
Ni—S2^i^	2.2075 (11)	C23—H23	0.93
Ni—S2	2.2075 (11)	C24—C25	1.394 (8)
S1—C1	1.731 (4)	C24—H24	0.93
S2—C1	1.743 (4)	C25—C26	1.383 (7)
S3—O2	1.427 (3)	C25—H25	0.93
S3—O1	1.434 (3)	C26—H26	0.93
S3—N1	1.629 (4)	C31—C32	1.381 (6)
S3—C2	1.766 (5)	C31—C36	1.382 (6)
N1—C1	1.294 (5)	C32—C33	1.391 (6)
C2—C3	1.517 (7)	C32—H32	0.93
C2—H2A	0.97	C33—C34	1.353 (7)
C2—H2B	0.97	C33—H33	0.93
C3—C4	1.507 (7)	C34—C35	1.367 (7)
C3—H3A	0.97	C34—H34	0.93
C3—H3B	0.97	C35—C36	1.392 (6)
C4—C5	1.537 (9)	C35—H35	0.93
C4—H4A	0.97	C36—H36	0.93
C4—H4B	0.97	C41—C46	1.396 (6)
C5—C6	1.543 (11)	C41—C42	1.400 (6)
C5—H5A	0.97	C42—C43	1.370 (6)
C5—H5B	0.97	C42—H42	0.93
C6—C7	1.534 (17)	C43—C44	1.376 (6)
C6—H6A	0.97	C43—H43	0.93
C6—H6B	0.97	C44—C45	1.388 (7)
C7—C8	1.538 (17)	C44—H44	0.93
C7—H7A	0.97	C45—C46	1.367 (6)
C7—H7B	0.97	C45—H45	0.93
C8—C9	1.536 (18)	C46—H46	0.93
C8—H8A	0.97	C11—C16	1.395 (6)
C8—H8B	0.97	C11—C12	1.399 (6)
C9—H9A	0.96	C12—C13	1.377 (6)
C9—H9B	0.96	C12—H12	0.93
C9—H9C	0.96	C13—C14	1.377 (7)
P1—C11	1.792 (4)	C13—H13	0.93
P1—C41	1.793 (4)	C14—C15	1.362 (7)
P1—C21	1.796 (4)	C14—H14	0.93
P1—C31	1.806 (4)	C15—C16	1.399 (6)
C21—C22	1.378 (6)	C15—H15	0.93
C21—C26	1.385 (6)	C16—H16	0.93
C22—C23	1.386 (7)		
			
S1—Ni—S1^i^	175.66 (8)	C22—C21—C26	120.2 (4)
S1—Ni—S2^i^	101.31 (4)	C22—C21—P1	121.6 (3)
S1^i^—Ni—S2^i^	78.52 (4)	C26—C21—P1	117.6 (3)
S1—Ni—S2	78.52 (4)	C21—C22—C23	118.9 (5)
S1^i^—Ni—S2	101.31 (4)	C21—C22—H22	120.5
S2^i^—Ni—S2	175.75 (8)	C23—C22—H22	120.5
C1—S1—Ni	87.00 (15)	C24—C23—C22	121.4 (5)
C1—S2—Ni	86.61 (15)	C24—C23—H23	119.3
O2—S3—O1	116.2 (2)	C22—C23—H23	119.3
O2—S3—N1	113.0 (2)	C23—C24—C25	120.0 (5)
O1—S3—N1	105.9 (2)	C23—C24—H24	120
O2—S3—C2	108.7 (2)	C25—C24—H24	120
O1—S3—C2	107.2 (2)	C26—C25—C24	119.1 (5)
N1—S3—C2	105.1 (2)	C26—C25—H25	120.5
C1—N1—S3	123.6 (3)	C24—C25—H25	120.5
N1—C1—S1	121.2 (3)	C25—C26—C21	120.4 (5)
N1—C1—S2	131.7 (4)	C25—C26—H26	119.8
S1—C1—S2	107.0 (2)	C21—C26—H26	119.8
C3—C2—S3	112.7 (3)	C32—C31—C36	120.1 (4)
C3—C2—H2A	109.1	C32—C31—P1	117.1 (3)
S3—C2—H2A	109.1	C36—C31—P1	122.6 (3)
C3—C2—H2B	109.1	C31—C32—C33	119.2 (4)
S3—C2—H2B	109.1	C31—C32—H32	120.4
H2A—C2—H2B	107.8	C33—C32—H32	120.4
C4—C3—C2	112.4 (4)	C34—C33—C32	120.4 (5)
C4—C3—H3A	109.1	C34—C33—H33	119.8
C2—C3—H3A	109.1	C32—C33—H33	119.8
C4—C3—H3B	109.1	C33—C34—C35	121.2 (4)
C2—C3—H3B	109.1	C33—C34—H34	119.4
H3A—C3—H3B	107.9	C35—C34—H34	119.4
C3—C4—C5	113.4 (5)	C34—C35—C36	119.5 (5)
C3—C4—H4A	108.9	C34—C35—H35	120.3
C5—C4—H4A	108.9	C36—C35—H35	120.3
C3—C4—H4B	108.9	C31—C36—C35	119.6 (4)
C5—C4—H4B	108.9	C31—C36—H36	120.2
H4A—C4—H4B	107.7	C35—C36—H36	120.2
C4—C5—C6	103.8 (6)	C46—C41—C42	118.8 (4)
C4—C5—H5A	111	C46—C41—P1	122.1 (3)
C6—C5—H5A	111	C42—C41—P1	119.1 (3)
C4—C5—H5B	111	C43—C42—C41	120.2 (4)
C6—C5—H5B	111	C43—C42—H42	119.9
H5A—C5—H5B	109	C41—C42—H42	119.9
C7—C6—C5	105.2 (7)	C42—C43—C44	120.5 (4)
C7—C6—H6A	110.7	C42—C43—H43	119.7
C5—C6—H6A	110.7	C44—C43—H43	119.7
C7—C6—H6B	110.7	C43—C44—C45	119.7 (4)
C5—C6—H6B	110.7	C43—C44—H44	120.1
H6A—C6—H6B	108.8	C45—C44—H44	120.1
C6—C7—C8	115.7 (9)	C46—C45—C44	120.4 (4)
C6—C7—H7A	108.4	C46—C45—H45	119.8
C8—C7—H7A	108.4	C44—C45—H45	119.8
C6—C7—H7B	108.4	C45—C46—C41	120.3 (4)
C8—C7—H7B	108.4	C45—C46—H46	119.8
H7A—C7—H7B	107.4	C41—C46—H46	119.8
C9—C8—C7	129.8 (11)	C16—C11—C12	119.1 (4)
C9—C8—H8A	104.8	C16—C11—P1	120.8 (3)
C7—C8—H8A	104.8	C12—C11—P1	120.1 (3)
C9—C8—H8B	104.8	C13—C12—C11	119.8 (4)
C7—C8—H8B	104.8	C13—C12—H12	120.1
H8A—C8—H8B	105.8	C11—C12—H12	120.1
C8—C9—H9A	109.5	C12—C13—C14	120.5 (5)
C8—C9—H9B	109.5	C12—C13—H13	119.8
H9A—C9—H9B	109.5	C14—C13—H13	119.8
C8—C9—H9C	109.5	C15—C14—C13	121.0 (4)
H9A—C9—H9C	109.5	C15—C14—H14	119.5
H9B—C9—H9C	109.5	C13—C14—H14	119.5
C11—P1—C41	108.68 (18)	C14—C15—C16	119.5 (4)
C11—P1—C21	111.12 (19)	C14—C15—H15	120.2
C41—P1—C21	106.86 (19)	C16—C15—H15	120.2
C11—P1—C31	108.82 (19)	C11—C16—C15	120.1 (4)
C41—P1—C31	113.36 (18)	C11—C16—H16	119.9
C21—P1—C31	108.01 (18)	C15—C16—H16	119.9
			
S2^i^—Ni—S1—C1	169.45 (15)	C11—P1—C31—C36	−99.4 (4)
S2—Ni—S1—C1	−6.21 (15)	C41—P1—C31—C36	21.7 (4)
S1—Ni—S2—C1	6.17 (15)	C21—P1—C31—C36	139.9 (4)
S1^i^—Ni—S2—C1	−169.41 (15)	C36—C31—C32—C33	2.3 (7)
O2—S3—N1—C1	54.5 (5)	P1—C31—C32—C33	−174.3 (4)
O1—S3—N1—C1	−177.2 (4)	C31—C32—C33—C34	−0.4 (8)
C2—S3—N1—C1	−63.9 (4)	C32—C33—C34—C35	−1.1 (8)
S3—N1—C1—S1	174.3 (2)	C33—C34—C35—C36	0.9 (8)
S3—N1—C1—S2	−2.7 (7)	C32—C31—C36—C35	−2.5 (7)
Ni—S1—C1—N1	−169.6 (4)	P1—C31—C36—C35	173.9 (4)
Ni—S1—C1—S2	8.07 (19)	C34—C35—C36—C31	0.9 (8)
Ni—S2—C1—N1	169.3 (5)	C11—P1—C41—C46	66.4 (4)
Ni—S2—C1—S1	−8.06 (19)	C21—P1—C41—C46	−173.6 (3)
O2—S3—C2—C3	65.5 (4)	C31—P1—C41—C46	−54.8 (4)
O1—S3—C2—C3	−60.8 (5)	C11—P1—C41—C42	−112.2 (3)
N1—S3—C2—C3	−173.2 (4)	C21—P1—C41—C42	7.8 (4)
S3—C2—C3—C4	−175.9 (4)	C31—P1—C41—C42	126.7 (3)
C2—C3—C4—C5	73.1 (6)	C46—C41—C42—C43	0.3 (6)
C3—C4—C5—C6	177.4 (6)	P1—C41—C42—C43	179.0 (3)
C4—C5—C6—C7	176.9 (10)	C41—C42—C43—C44	1.1 (6)
C5—C6—C7—C8	−179.6 (12)	C42—C43—C44—C45	−1.4 (7)
C6—C7—C8—C9	−5(3)	C43—C44—C45—C46	0.2 (7)
C11—P1—C21—C22	17.5 (4)	C44—C45—C46—C41	1.2 (7)
C41—P1—C21—C22	−100.9 (4)	C42—C41—C46—C45	−1.5 (6)
C31—P1—C21—C22	136.8 (3)	P1—C41—C46—C45	179.9 (3)
C11—P1—C21—C26	−171.4 (3)	C41—P1—C11—C16	2.8 (4)
C41—P1—C21—C26	70.1 (4)	C21—P1—C11—C16	−114.5 (4)
C31—P1—C21—C26	−52.1 (4)	C31—P1—C11—C16	126.7 (3)
C26—C21—C22—C23	−1.1 (7)	C41—P1—C11—C12	−176.3 (3)
P1—C21—C22—C23	169.7 (4)	C21—P1—C11—C12	66.4 (4)
C21—C22—C23—C24	0.2 (7)	C31—P1—C11—C12	−52.4 (4)
C22—C23—C24—C25	0.4 (8)	C16—C11—C12—C13	−1.4 (6)
C23—C24—C25—C26	−0.1 (8)	P1—C11—C12—C13	177.8 (3)
C24—C25—C26—C21	−0.8 (8)	C11—C12—C13—C14	1.4 (7)
C22—C21—C26—C25	1.4 (7)	C12—C13—C14—C15	−1.0 (7)
P1—C21—C26—C25	−169.7 (4)	C13—C14—C15—C16	0.5 (7)
C11—P1—C31—C32	77.1 (4)	C12—C11—C16—C15	0.9 (6)
C41—P1—C31—C32	−161.9 (3)	P1—C11—C16—C15	−178.3 (3)
C21—P1—C31—C32	−43.6 (4)	C14—C15—C16—C11	−0.5 (7)

Symmetry codes: (i) −*x*, *y*, −*z*+1/2.

### Hydrogen-bond geometry (Å, °)

**Table:**

*D*—H···*A*	*D*—H	H···*A*	*D*···*A*	*D*—H···*A*
C2—H2B···S2	0.97	2.83	3.490 (5)	126
C13—H13···O2^ii^	0.93	2.58	3.276 (6)	132

Symmetry codes: (ii) −*x*+1/2, *y*−1/2, −*z*+1/2.
